# Innovations in reagent manufacturing to support the global diagnostics supply chain

**DOI:** 10.3389/fpubh.2026.1899854

**Published:** 2026-08-31

**Authors:** Anna R. Bird, Jacqueline C. Linnes, Laud Anthony Basing, Elizabeth A. H. Hall

**Affiliations:** 1 Imperial Global Singapore, Imperial College London, London, United Kingdom; 2 Department of Chemical Engineering and Biotechnology, University of Cambridge, Cambridge, United Kingdom; 3 Weldon School of Biomedical Engineering, Purdue University, West Lafayette, IN, United States; 4 Department of Medical Diagnostics, Kwame Nkrumah University of Science and Technology, Kumasi, Ghana

**Keywords:** distributed manufacturing, global health equity, low-cost diagnostics, molecular diagnostics, technology transfer

## Abstract

Diagnostics are not only tools for clinical decision making, but also drivers of the healthcare supply chain. They quantify and monitor the prevalence of disease across global populations. At high levels, this population health data is used to inform the purchasing and distribution of health care goods. There is overwhelming evidence that access to diagnostics is limited in low-and middle-income countries. This is true for both in-vitro molecular diagnostics and diagnostic imaging, but the scope of this work is limited to the former. While the factors contributing to limited access are numerous, interconnected, and complex, we propose a method to alleviate some restraints within the supply chain: distributed and local manufacturing of key biological reagents, or active diagnostics ingredients (ADIs). In addition to shifting the geographical boundaries of manufacturing and moving goods closer to areas of deployment, this could reduce global power imbalances by redistributing ownership of diagnostics goods, reagents, and intellectual property.

## 1 Introduction

In low- and middle-income countries (LMICs), infectious and neglected tropical diseases (NTDs) impact health and productivity. Late diagnosis and inadequate management results in poorer disease outcome. A correlation has been made between rise in certain NTDs and reduction in economic productivity, as measured through Gross Domestic Product (GDP) (1). Where this disease burden is highest and the cost barrier is most limiting, *in-vitro* diagnostics (IVD) are often not accessible (2). Accurate testing is crucial for clinical decision making.

While nucleic acid molecular diagnostics have shown improved performance over traditional immunoassays, their implementation in low- and middle-income countries (LMICs) is often limited by the high cost of reagents (3) cold chain storage requirements, and the need for technical expertise (4). There is still widespread scarcity of diagnostic capacity, with the most severe gap occurring at the level of primary care, in which only 19% of populations in LMICs had access to the simplest of diagnostic tests (5).

While the COVID-19 pandemic saw a rise in manufacturing capacity globally, many of the pipelines have since been dismantled (6). Investment across the production pathway, including training of personnel, R&D, and procurement of raw materials is necessary for sustained manufacturing capacity. Many manufacturing centers in LMICs operate a fill and finish packaging and labelling with limited upstream production (7). The use of emerging manufacturing models for biological reagents shifts the focus to upstream goods, which have cross-application uses. The biological reagents, which are shared among many diagnostic assays, are the active diagnostic ingredients (ADIs). Local manufacturing, which occurs close to the point-of-need, domestic manufacturing, which occurs at a national level to circumvent import challenges, and on-demand manufacturing, which can be scaled up or down in response to need, must play a greater role in supplementing key existing economic models.

## 2 Diagnostics: barriers to access

At the peak of the COVID-19 pandemic, production and shipping of critical reagents, such as enzymes, primers, paper-based membranes, and general lab supplies were disrupted (8, 9). Despite strong technical capabilities available, researchers and test developers in LMICs were stymied by supply chain shortages that left them without enzymes, primers, and membranes for nucleic acid amplification tests (NAATs). The COVID-19 pandemic demonstrated the limits of global supply chains and capacity for in-country and regional manufacturing across the spectrum of test development, from biochemical reagents to large-scale manufacturing are needed. Such times of extreme need also prompted high income countries (HICs) to prioritize domestic populations at the expense of global distribution (10).

The cost of reagents is determined by the limited number of companies authorized to produce and distribute reagents for diagnostic purposes. HICs dominate the global supply of diagnostics, resulting in revenue generation in HICs for IVDs which are increasingly utilised in LMICs. The democratisation of diagnostics requires involvement of local communities in “developing innovative solutions for their own problems relating to health-care delivery” (5). Diagnostics and pharmaceutical companies calculate the ‘net present value’ of one disease target versus another, which systematically devalues diseases that are endemic in LMICs and thus offer lesser financial returns (11, 12).

A heavy reliance on a trickle-down model can result in LMICs waiting for more than a decade to receive products and innovations from HICs, which were developed without cognizance of geographical differences in disease biology and healthcare logistics (5). The production of POC diagnostics in LMICs has been proposed as an essential technology fit for a low-resource area and supply chain sustainability (13). The rise in production to meet this need during emergency situations demonstrated during the COVID-19 pandemic demonstrates that this is technically feasible, but the sharp decline at the end of the pandemic highlights a need to consider economic models which make this sustainable long term (6).

Technology transfer becomes cost prohibitive when the expense of importing reagents and materials (the ‘bill of materials’, BOM) is considered (14). To achieve long-term socioeconomic independence, the local supply chain needs to include even the basic active reagents and materials. Furthermore, following a traditional production model, production scale is a significant disadvantage, as sufficient economies of scale are required to meet target price range (14). For over two decades sub-Saharan Africa has highlighted the need to achieve synergies between expansion of a local manufacturing infrastructure and socio-economic long-term independence from external grant-in-aid to supply diagnostics.

## 3 The role of diagnostics and reagents in the global health supply chain

Diagnostics go beyond identifying health needs at the point of care, as they are also used for real-time epidemiological monitoring and health forecasting of both chronic and infectious diseases. Restricted access to affordable diagnostics impacts robust information in the health systems regarding NTD outbreaks and clinical management needs and thereby has a resultant effect on procurement and supply of other critical healthcare products (15).

**Figure 1:**
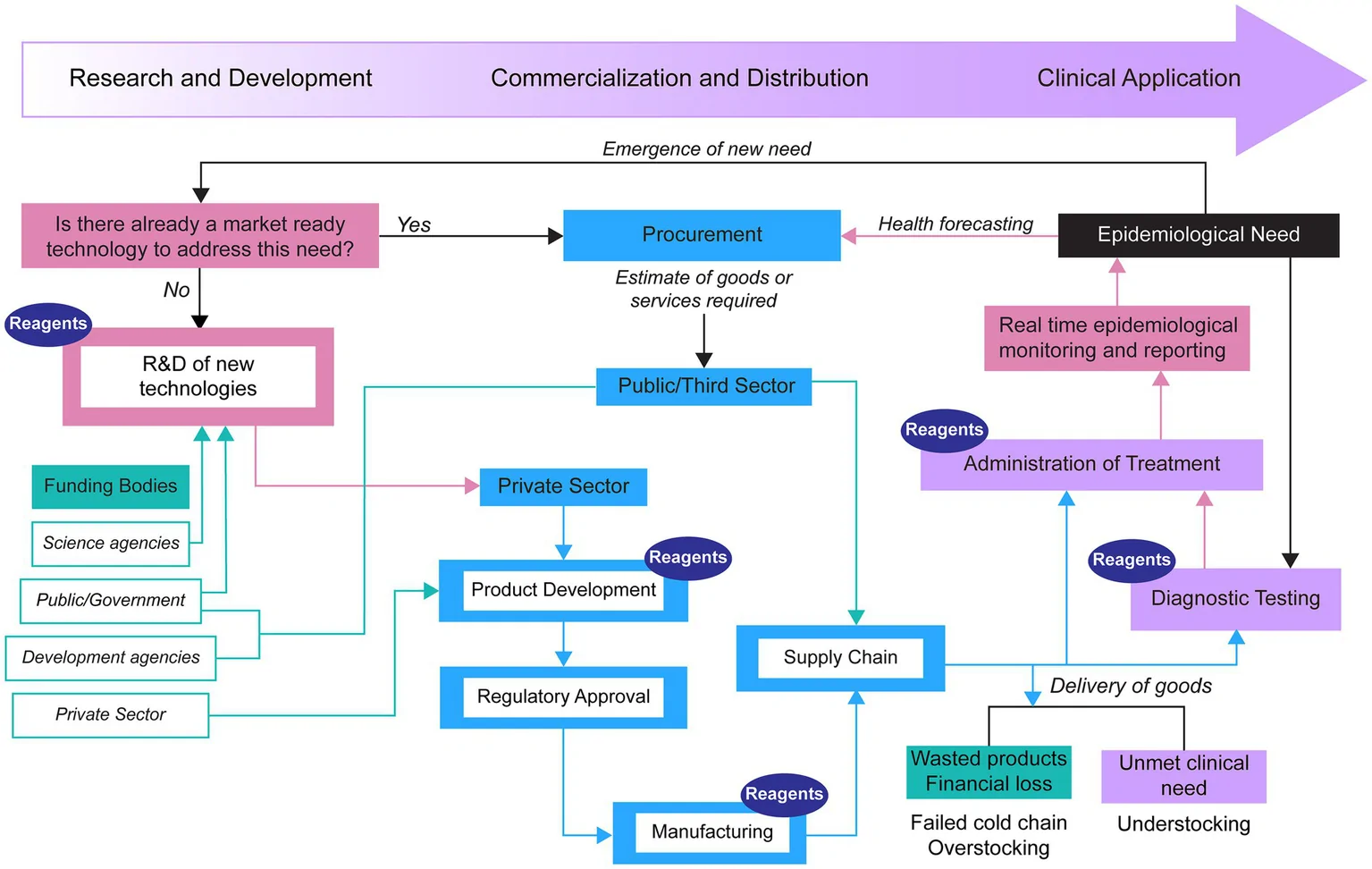
Overview of the flow of goods and services (blue), knowledge (pink), and funding (green) in global healthcare toward meeting a clinical need (purple). Areas where molecular biology reagents are utilized are indicated with a dark blue circle. Reagent applications span the entire diagnostics pipeline, from R&D of new technologies, to product development and deployment in diagnostic assays. Leveraging the continued demand of biological reagents for R&D and product development can sustain manufacturing capacity during non-emergency periods. A diagram with expanded detail is included in Supplementary Figure 1.

Figure 1 demonstrates the interconnectedness of diagnostics in the global healthcare supply chain. Diagnostics identify the epidemiological need and inform the administration of treatment. As a need is identified, predictions are made on the number of diagnostics and therapeutics for procurement. Because there are many tiers between the clinical application and the procurement agents, there is a distortion of the data, even when assuming no financial barriers in the procurement pathway. As data is transmitted through the tiers, it becomes out of phase with real-time events, leading to large changes at the procurement level that do not map to the small changes in need at the clinical level (15).

The ability of governments to provide diagnostics or funding for diagnostics is also largely limited by the budget availability and the directive of external funders. Even in more resourced countries with a strong healthcare structure, unreliable and untimely data, as well as procurement decisions guided by social and political pressures, result in either understocking, leaving an unmet clinical need, or overstocking, resulting in financial loss as products go unused or expire during long storage times. Reliance on international supply chains also means long shipping times and customs delays, during which cold chain storage (when required) is likely to fail, causing even more product and monetary loss.

A macroscale view of the supply chains tends to focus on delivery of the end product – the diagnostic kit or the functionalised therapeutic – and overlooks the supply chain of components and materials. These include molecular biology reagents, which may be used directly as diagnostics or therapeutics or, from a microscale view of the supply chain, they may also be deployed at earlier steps – such as quality assurance testing during manufacturing, trialling design iterations during product development, or even experimenting at the earliest steps of Technology Innovation.

Figure 1 highlights the many points where molecular biology reagents are inputs to the healthcare product supply chain. At the level of clinical care, enzymes, for example tissue plasminogen activator (16), are deployed as therapies. These goods are developed and manufactured by processes which also rely on molecular biology reagents. Upstream of this, DNA polymerase enzymes may be deployed for research applications, such as PCR and DNA assembly – a first step in recombinantly producing proteins. Furthermore, DNA polymerases are a key element in nucleic acid diagnostics (17, 18). Nucleotides and their analogues are used in conjunction with DNA polymerases but can also have therapeutic applications. For example pyrimidine nucleoside analogue azidothymidine is used as a therapeutic in HIV management (19). This highlights the multiple applications for DNA amplification, from a tool for producing copies of a nucleic acid sequence for onward use in recombinant production of proteins, to a method for detecting nucleic acid sequences present as a biomarker of disease.

## 4 Innovative manufacturing to support diagnostic reagent needs

There is a need to move away from philanthropic funding of end product diagnostic devices and toward public and private investment in the diagnostic supply chain, including active diagnostic ingredients (ADIs) and raw materials on the BOM. To be sustainable, this must be supported by a socioeconomic model, where the impacts of employment, growth, and improved economic productivity emerge alongside the healthcare benefits. Distributed manufacturing is a trending topic, but deployment still tends to remain concentrated in HICs, where the original research was undertaken. National science agencies and public agencies are generally driven toward domestic issues, and early-stage research is highly investigator driven (20). The majority of this funding for research and development (R&D) is in the global north, perpetuating the lack of attention to neglected tropical diseases (7, 21). Development agencies also prefer to financially support operational and field research, leaving the gap of turning early-stage research into a market viable product, mostly to the private sector, which is more active outside of LMICs (21).

To address what has been called “the innovation starvation in public health” (21), domestic or on-demand manufacturing protocols have been proposed, which would aim to directly support scientists and R&D in LMICs. This may also be a strategy to reduce aid dependency and to support effective aid with sustainable outcome. To feed the innovation starvation, the rapidly growing capacity for innovation in LMICs must be utilised (22). This requires agencies to continue to support LMICs through sharing of goods and services, encouraging the development of local manufacturing, and knowledge dissemination via open-source methods (23) where appropriate. However, although open-source design, codes and solutions now contribute to numerous commercial products, they remain a part and not the whole of the solution. Both business and private investors are deterred by a lack of technology monopoly and there are tensions between the drive for open access (OA) research publication and IP protection, typically via patents (24), that make the necessary investment more challenging to monetarize. There are concerns over ownership and protection of intellectual property (IP). For example, participants in the WHO’s mRNA technology transfer programme felt they had little control over any IP they generated, as it was to become part of the IP pool owned by the consortium and be redistributed to other LMIC-based manufacturers (10). In LMICs financial investment and grant-aid need to focus on a sustainable supply chain model which is able to support global demand at affordable cost and become commercially self-supporting and revenue generating.

Several new manufacturing models have been proposed that will impact both research capability and delivery of healthcare and procurement of medical supplies in resource poor countries (25, 26). These include: (1) distributed manufacturing, where facilities are geographically dispersed, (2) local/domestic manufacturing, where goods are produced within close geographical proximity to the point of application, and (3) on-demand manufacturing, where the production of goods is carried out only when needed and in the quantities required. These models can work synergistically to overcome supply chain challenges, and each contributes unique solutions to the problem. Interest and support for new approaches to manufacturing in the healthcare sector is also underscored by the African union pledge for 60% of vaccines to be manufactured regionally by 2040 (27, 28).

**Figure 2:**
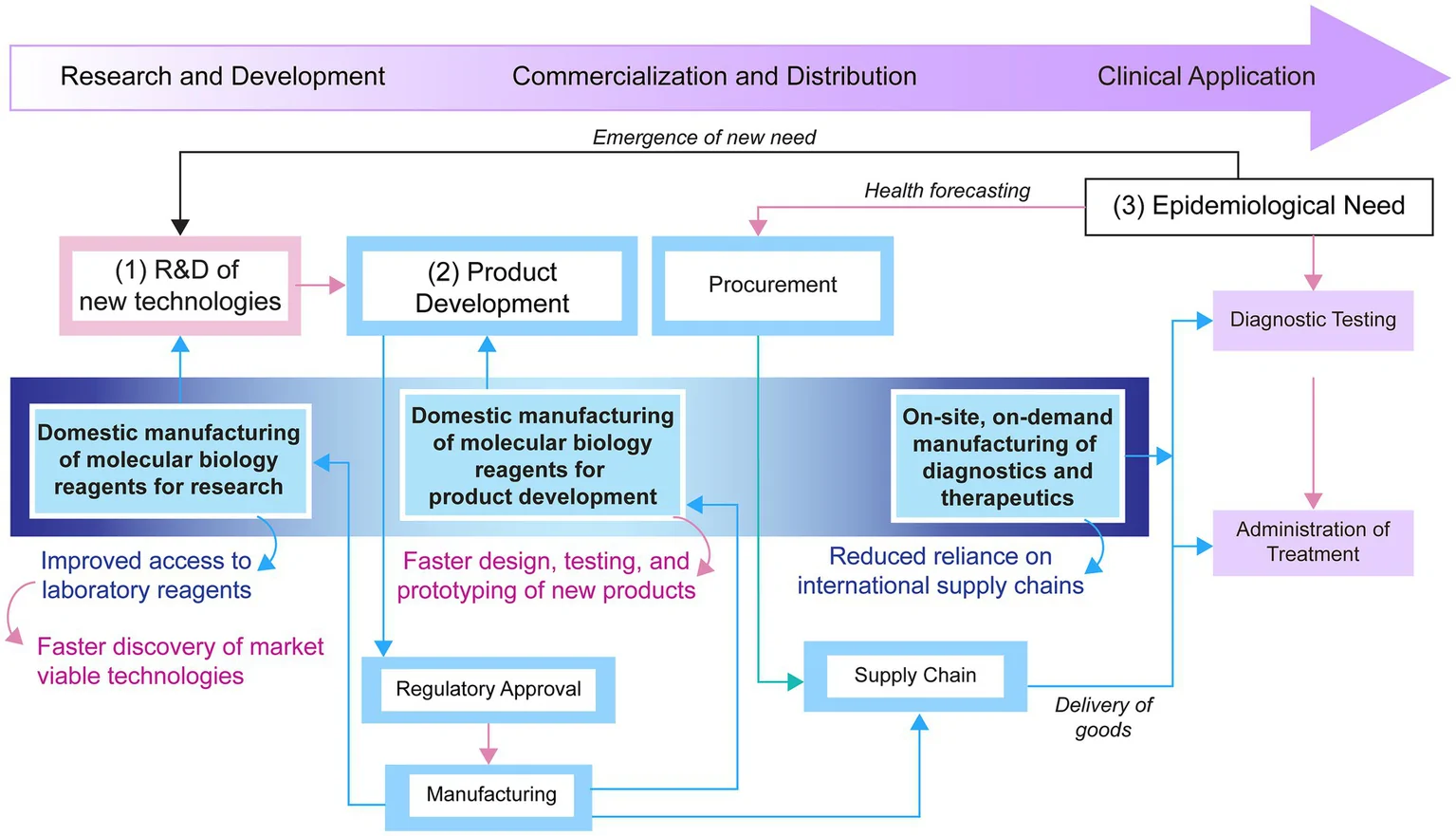
Global healthcare supply chains re-imagined with domestic and on-demand manufacturing. Domestic or local manufacturing are useful for meeting the demands of R&D and product development, promoting faster discovery of market viable technologies and prototypes of new products. On-site, on-demand manufacturing is useful for modulating the volume of production in response to fluctuations in the epidemiological need. A diagram with expanded detail is included in Supplementary Figure 2.

Figure 2 explores the impacts these manufacturing models could have on the complex healthcare supply chain. Domestic manufacturing is useful in that it bypasses the need for materials to clear customs; this already can reduce delays in receiving materials and improve the responsiveness of manufacturing to changes in local demand. On-site or local manufacturing goes one step further by taking the production of goods all the way to a city or regional level, which simplifies the problem of unnecessary complexity and long lead times between each tier (15). However, local manufacturing does not reduce cost if any of the raw materials must be imported at high cost from HICs, highlighting the need to develop synthesis pathways from accessible and renewable raw materials.

Even when good disease surveillance tools are available, population health conditions can change quickly. In the current centralised high volume manufacturing model, accurate product stocking requires health facilities to perform the “almost insurmountable task” of precisely forecasting their needs, particularly in the event of rapidly evolving conditions, such as a pandemic (15). The need for forecasting can be entirely circumvented by utilising on-demand synthesis. Additionally, as on-demand manufacturing enables real-time modulation of product volumes, it can reduce waste from overproduction. Biological goods often have a short shelf life, so on-demand synthesis is particularly useful in reducing the financial loss from expired goods, and cold-chain dependency leading to premature expiry, due to failed storage conditions like power cuts.

Because biological reagents are deployed at multiple points in the supply chain, local manufacturing would have a broad influence, both in supplying diagnostic kits and feeding into the R&D of new diagnostics and therapies, leading to a more equal global distribution of regionally relevant product-development, manufacturing and technology innovation, so long as there is an affordable supply chain of raw materials to feed it (Figure 2).

A primary concern in building manufacturing capacity of diagnostics is how to sustain volume during non-emergency periods when demand falls. However, diagnostics are required through the whole life-cycle from epidemic/pandemic preparedness to pathogen detection and management to contain spread, including (where available) monitoring vaccine efficacy (29, 30). Thus, programs of routine or rapid deployment of diagnostics are desirable in high-risk areas. Proposed solutions are to ensure ongoing diagnostic demand, for example through production of routine test kits for asymptomatic screening (31). Another strategy is cross-application of manufactured goods, such as deploying similar technologies for food and agricultural testing (6).

As the required biological reagents are not tied to a specific disease, this can help sustain demand, thus overcoming the challenge of collapse during non-emergency periods. Reagents such as DNA polymerases and nucleotides form the core of nucleic acid amplification and are common to all NAAT. The cross-application of reagents is detailed in Supplementary Table 1. By focusing on the manufacturing of reagents, rather than a fill and finish model of diagnostics, there is a sustained demand for the goods, which can be adapted to changes in the epidemiological need.

These reagents also have cross-applications in R&D, and in training programs, for example in the delivery of university level biotechnology courses, which also creates awareness and expertise at more regional levels. An important element of pandemic preparedness is continued investment by governments or international organizations in basic science education or in advanced biotechnology training (7). Beyond that, initiatives such as the African Union Migration policy framework aim to reduce brain drain and maintain skilled personnel on the continent (32).

## 5 Local production of nucleic acid amplification reagents

In response to supply chain limitations faced during the Covid-19 pandemic, many teams in LMICs partnered across continents with collaborators in HICs via consortia such as AfriDx (33) and ReClone (34), to build capacity to produce enzymes for NAATs, tailored for low cost local production or to develop open sourced reagents, respectively (35). Emerging government-led initiatives are seeking greater local autonomy in manufacturing of both the end-product as well as ADIs. One example is the Oswaldo Cruz Foundation (Fiocruz) in Brazil, which announced the production of active diagnostic ingredients (ADIs) for reverse-transcription polymerase chain reaction (RT-PCR) tests for Covid-19 and Influenza (36).

There are several other published examples of local or in-house production of biological reagents, that suggest that translation to local manufacturing is increasingly viable, both technically and affordably. Locally produced enzymes have been deployed in loop-mediated isothermal amplification (LAMP) in diagnosing malaria in clinical samples in Ghana (37) and RT-LAMP in identifying the DENV-1 serotype for Dengue outbreaks (38). The Covid-19 pandemic has also shown that the enzymes for RT-LAMP can be successfully produced in-house locally without the need for import of these vital reagents (39) and Mote *et al.* have created a PCR master mix with reagents produced in-house (40). Additionally, nucleic acid amplification requires 2′-deoxynucleoside 5′-triphosphates (dNTPs), the building blocks for the synthesis of the amplified DNA. Methods for production of dNTPs from bacterial genomic DNA, a readily available and regenerable starting material, have also been demonstrated (41, 42).

The emerging manufacturing techniques rely on renewable or regenerable starting materials. Once initial investment in incubators, centrifuges, and cell lines is made, the cost of recombinantly producing proteins (ie DNA polymerases) in *E. coli* or yeast is dependent primarily on the cost of cell culture media and purification. To eliminate the need for expensive centrifuges and their required maintenance, solid state cell culture has been proposed (43). Further, novel purification techniques use *in vivo* fused affinity tags. Protein engineering enables modular designs, where this affinity tag can be changed depending on what raw materials are available. For example, a silica affinity tag has been proposed as fit for purpose where high silica content sand is a natural resource (44). Further improvements in the manufacturing technology should focus on reducing reliance on expensive equipment, which often requires technicians from outside LMICs for servicing due to lack of local expertise (45), and the use of readily available or regenerable starting materials.

These case studies demonstrate the technical feasibility of local, domestic, or on-demand manufacturing. However, this does not necessarily provide evidence that the reagent production can be maintained at-scale over time. Success requires synergistic implementation of these technologies with existing economic models and strategic investment strategies. For example, pooled procurement can support affordable procurement of dry goods, such as the salts and chemicals which form the basis of cell culture media and buffers. These goods have a long shelf life and are not temperature sensitive. Local and domestic manufacturing facilities should work in tandem with regional manufacturing hubs. Local and domestic manufacturing of biological reagents for cross-application use in research markets should sustain manufacturing capacity and infrastructure during low demand periods, so that on-demand manufacturing facilities are available for surges in demand.

Investment is required to strengthen the diagnostics sector in LMICs. Donor funds are an inadequate model to meet pandemic needs, and there is chronic underinvestment in global R&D infrastructure (7). A sustainable financing mechanism to support training, R&D, reagent manufacturing, and diagnostics production requires coordinated action across the global financial system (46). Public investment should be complemented by private sector engagement, and strategies are needed to derisk investment in manufacturing capacity (6).

## 6 Regulatory processes for *in vitro* diagnostic approved use

For reagents to be used as part of a diagnostic kit, or for a locally produced diagnostic kit to be deployed, the goods must be subject to quality assurance and regulatory approval. The regulatory environment varies greatly across LMICs. Many countries have no legal framework establishing regulatory environments and often depend on regulatory processes and assessments from other countries or the World Health Organization (WHO). This complexity only increases in the early stages of an evolving pandemic while dealing with highly variable uncertainties in clinical, political, and biosafety standpoints. This was the case during Covid-19 and will remain true for pandemics caused by emerging pathogens in the future. Designing regulatory pathways which are responsive to the dynamics of infectious disease will be a critical element of pandemic preparedness. The WHO prequalification pathway for *in vitro* diagnostics (PQDx) and Global Model Regulatory Framework for Medical Devices including in vitro diagnostics are steps toward resolution. However, PQDx is only available to targets on the WHO-IVD eligibility list (47), and the products must be commercially available at the time of submission. Since PQDx is focused on the “original legal manufacturer,” it favors the ‘fill and finish’ model of diagnostics, rather than *de novo* regional manufacturing. In some LMICs, a European Conformity (CE) mark on a diagnostic product is enough for it to be accepted for use. In countries that rely on approvals from external sources, there is currently no pathway to get approval for locally produced products – and thus no incentive for technology development. Having government support and independent regulatory approval pathways is thus also an important step for enabling domestic IVD manufacturing and maintaining a quality control pathway. Almost all countries with highly developed regulatory environments insisted on the use of the PCR as its gold standard whilst giving approval for the use of antibody and antigen tests for specific professional purposes (48, 49).

High quality, reliable goods which meet regulatory requirements are critical to ensuring continued investment in LMIC manufacturing, and to building public trust in domestically and locally produced goods. Initiatives such as the Africa Collaborative Initiative to Advance Diagnostics (AFCAD) are working to harmonize regulatory systems on the continent, build laboratory centers of excellence, and establish a network of accredited laboratories for IVD validation (50).

## 7 Conclusion

Significant barriers to accessing diagnostics are cost, cold-chain storage requirements, and dependence on grant-aid money and imported devices from HICs. Beyond individual diagnosis and treatment, IVDs are also important for disease surveillance, including both infectious and noncommunicable disease. Diagnostics inform the healthcare supply chain by transmitting critical information about population health, which is used in making decisions on the procurement of healthcare goods. Innovative manufacturing methods, such as domestic and on-demand manufacturing have been proposed as technologies fit for purpose, and there are examples of effective domestic manufacturing for diagnostics, including the AfriDx (33) and ReClone (34) consortia, as well as the Oswaldo Cruz Foundation (Fiocruz) (36). Emerging initiatives should also seek to include CRISPR enzymes, which are expected to play an important role in the next generation of molecular diagnostics (51).

While domestic manufacturing does not guarantee stability in supply, it helps keep money in the economy, employs people, and invests in infrastructure. Further, the African trade union argues that domestic production will aid in the development of industrial and technical infrastructure to enhance long-term health security, self-sufficiency, and employment (14). There are concerns that local manufacturers will not be able to match donor prices, but to move toward a self-sustainable healthcare system, LMICs must reduce over-reliance from donor support (13). Beyond the critical need for reagents to perform diagnostic testing is the requirement for reagents to innovate novel diagnostics.

Local, domestic, and on-demand manufacturing of biological reagents could be integral in building a resilient diagnostic pipeline, but only if embedded in a sustainable economic, regulatory, and procurement framework. Policy-driven incentives can support the uptake of technologies which have already been demonstrated as feasible. Through local and on-demand manufacturing of reagents, the materials which underpin research and development of IVDs will be within reach and responsive to LMIC health needs.

## Data Availability

The original contributions presented in the study are included in the article/Supplementary material, further inquiries can be directed to the corresponding author/s.
