# Supplementary material for: Innovations in reagent manufacturing to support the global diagnostics supply chain
Source: Front Public Health. 2026 Aug 31;14:1899854. doi: 10.3389/fpubh.2026.1899854 (PMC13572216; doi:10.3389/fpubh.2026.1899854)
Supplement: Supplementary file 1 [file Data_Sheet_1.DOCX]

Innovations in reagent manufacturing to support the global diagnostics supply chain: Supplementary Material

**Anna R Bird^1,2 *^, Jacqueline C Linnes^3^, Laud Anthony Basing^4^, Elizabeth A H Hall^2 *^**

^1^Imperial Global Singapore, Imperial College London, London, UK, SW7 2AZ, ORCID: https://orcid.org/0000-0003-0354-8824

^2^Department of Chemical Engineering and Biotechnology, University of Cambridge, Cambridge, UK, CB3 0AS, ORCID: https://orcid.org/0000-0001-9572-9854

*^3^*Weldon School of Biomedical Engineering, Purdue University, West Lafayette, IN 47907, USA, ORCID: https://orcid.org/0000-0003-4962-0908

^4^Department of Medical Diagnostics, Kwame Nkrumah University of Science and Technology, Kumasi, Ghana, ORCID: https://orcid.org/0000-0001-7112-1123

*** Correspondence:**
*Primary corresponding author: Anna R Bird ([a.bird@imperial.ac.uk](mailto:a.bird@imperial.ac.uk))

*Corresponding author: Elizabeth A H Hall ([eah16@cam.ac.uk](mailto:eah16@cam.ac.uk))

**Supplementary Figure 1.** Overview of the flow of goods, services, knowledge, and funding in global healthcare. The arrows show relative time scales, where a solid line is the fastest, and longer dashes indicate slower flow. The boxes for product development, regulatory approval, and technology innovation show relative contributions by the global north and the global south.

**Supplementary Figure 2.** Expanded view of the role of domestic, local, and on-demand manufacturing in (1) R&D, (2) Product Development, and (3) Epidemiological need.

**Supplementary Table 1.** Cross-applications of molecular biology reagents

|  | Research Applications | Diagnostic Applications | Therapeutic Applications |
| --- | --- | --- | --- |
| Proteins | | | |
| Enzymes | DNA polymerases:  PCR for DNA cloning and assembly (1)  HRP and ALP:  Colorimetric readout of assays (ELISA) (2) | Taq and Bst DNA polymerases:  NAAT (PCR, LAMP) (3,4)  Glucose oxidase, glucose dehydrogenase, and hexokinase:  glucose meters (5) | Tissue plasminogen activator (tPA):  thrombolytic drug (6) |
| Antibodies | Primary and secondary labelling antibodies:  ELISA and Western Blot (2) | Capture antibodies:  Lateral flow tests | Therapeutic antibodies:  oncology, immunology, and haematology applications (7) |
| Small Molecules | | | |
| Nucleotides and nucleotide analogues | dNTPs:  PCR for DNA cloning and assembly (1)  dNTPs/ddNTPs:  Sequencing (8) | dNTPs:  NAAT (PCR, LAMP) (3,4) | Azidothymidine, pyrimidine nucleoside analogue:  HIV treatment (9) |

**References**

1.	Hughes RA, Ellington AD. Synthetic DNA Synthesis and Assembly: Putting the Synthetic in Synthetic Biology. Cold Spring Harb Perspect Biol. 2017 Jan;9(1):a023812. doi:10.1101/cshperspect.a023812 PubMed PMID: 28049645; PubMed Central PMCID: PMC5204324.

2.	Beyzavi K, Hampton S, Kwasowski P, Fickling S, Marks V, Clift R. Comparison of Horseradish Peroxidase and Alkaline Phosphatase-Labelled Antibodies in Enzyme Immunoassays. Ann Clin Biochem. 1987 Mar 1;24(2):145–52. doi:10.1177/000456328702400204

3.	Boehm CD. Use of polymerase chain reaction for diagnosis of inherited disorders. Clin Chem. 1989;35(9):1843–8. doi:10.1093/clinchem/35.9.1843 PubMed PMID: 2570652.

4.	Wong YP, Othman S, Lau YL, Radu S, Chee HY. Loop-mediated isothermal amplification (LAMP): a versatile technique for detection of micro-organisms. J Appl Microbiol. 2018;124(3):626–43. doi:10.1111/jam.13647

5.	Tonyushkina K, Nichols JH. Glucose Meters: A Review of Technical Challenges to Obtaining Accurate Results. J Diabetes Sci Technol. 2009 Jul 1;3(4):971–80. doi:10.1177/193229680900300446

6.	Vachher M, Sen A, Kapila R, Nigam A. Microbial therapeutic enzymes: A promising area of biopharmaceuticals. Curr Res Biotechnol. 2021 Jan 1;3:195–208. doi:10.1016/j.crbiot.2021.05.006

7.	Lu RM, Hwang YC, Liu IJ, Lee CC, Tsai HZ, Li HJ, et al. Development of therapeutic antibodies for the treatment of diseases. J Biomed Sci. 2020 Jan 2;27(1):1. doi:10.1186/s12929-019-0592-z

8.	Sanger F, Nicklen S, Coulson AR. DNA sequencing with chain-terminating inhibitors. Proc Natl Acad Sci. 1977 Dec;74(12):5463–7. doi:10.1073/pnas.74.12.5463

9.	Lavie A, Ostermann N, Brundiers R, Goody RS, Reinstein J, Konrad M, et al. Structural basis for efficient phosphorylation of 3′-azidothymidine monophosphate by Escherichia coli thymidylate kinase. Proc Natl Acad Sci. 1998 Nov 24;95(24):14045–50. doi:10.1073/pnas.95.24.14045
